# Ecological, (epi)genetic and physiological aspects of bet-hedging in angiosperms

**DOI:** 10.1007/s00497-020-00402-z

**Published:** 2021-01-15

**Authors:** Maraeva Gianella, Kent J. Bradford, Filippo Guzzon

**Affiliations:** 1 Department of Biology and Biotechnology “L. Spallanzani”, University of Pavia, 27100 Pavia, Italy; 2 Department of Plant Sciences, Seed Biotechnology Center, University of California, Davis, USA; 3 International Maize and Wheat Improvement Center (CIMMYT), Carretera México-Veracruz, Km. 45, El Batán, 56237 Texcoco, Mexico State Mexico

**Keywords:** Bet-hedging, Heteromorphism, Fitness, Seed dormancy, Eco-physiology, Soil seed bank

## Abstract

Bet-hedging is a complex evolutionary strategy involving morphological, eco-physiological, (epi)genetic and population dynamics aspects. We review these aspects in flowering plants and propose further research needed for this topic.

Bet-hedging is an evolutionary strategy that reduces the temporal variance in fitness at the expense of a lowered arithmetic mean fitness. It has evolved in organisms subjected to variable cues from the external environment, be they abiotic or biotic stresses such as irregular rainfall or predation. In flowering plants, bet-hedging is exhibited by hundreds of species and is mainly exerted by reproductive organs, in particular seeds but also embryos and fruits. The main example of bet-hedging in angiosperms is diaspore heteromorphism in which the same individual produces different seed/fruit morphs in terms of morphology, dormancy, eco-physiology and/or tolerance to biotic and abiotic stresses in order to ‘hedge its bets’ in unpredictable environments. The objective of this review is to provide a comprehensive overview of the ecological, genetic, epigenetic and physiological aspects involved in shaping bet-hedging strategies, and how these can affect population dynamics. We identify several open research questions about bet-hedging strategies in plants: 1) understanding ecological trade-offs among different traits; 2) producing more comprehensive phylogenetic analyses to understand the diffusion and evolutionary implications of this strategy; 3) clarifying epigenetic mechanisms related to bet-hedging and plant responses to environmental cues; and 4) applying multi-omics approaches to study bet-hedging at different levels of detail. Clarifying those aspects of bet-hedging will deepen our understanding of this fascinating evolutionary strategy.

## Introduction

Organisms must cope with a variety of threats to their survival, ranging from abiotic stresses, such as the lack of resources or unfavourable climatic conditions, to biotic stresses such as predation and infections. Plants, being sessile organisms, evolved different strategies to overcome these environmental factors, depending on their temporal variability. When environmental variation is consistent, such as seasonal regularity, physiological or developmental plasticity is sufficient to allow individuals to adapt their phenotypes in response to the prevailing conditions. When, on the contrary, environmental variation is unpredictable, organisms apply diverse options without predicting how it will affect their future fitness, thereby ‘hedging their bets’ (Childs et al. 2010; Slatkin 1974). Bet-hedging occurs when a population lowers its mean fitness over time (across years) by decreasing also its annual variance in survival, thus improving its long-term fitness (Olofsson et al. 2009; Seger and Brockmann 1987). The canonical example of bet-hedging in plants is seed dormancy, i.e. the temporary failure of a seed to complete germination under favourable conditions (Bewley et al. 2013). When seeds germinate, the probability of survival for the seedlings is heavily dependent on the environmental conditions at that time. If a dry period occurs, the seedling is almost certainly bound to perish, while, if germination occurs during a wet period, survival is more likely. When the occurrence of dry/wet periods is not predictable, seed dormancy allows a distribution of germination events that, just like placing bets, enhances the probability that a proportion of the seed cohort germinates during a wet period (Cohen 1966). When different seed phenotypes are produced at the same time, only the ones adapted to the ongoing conditions survive. This way, survival of part of the progeny is assured, even if the mean fitness of the mother plant for that particular timeframe is not at its maximum (here referred to the maximum fitness possible, when a high proportion of the seeds produced survive because of specialized adaptation to that particular condition). Indeed, bet-hedging strategies tend to lower the arithmetic mean of fitness of single generations, at the same time lowering the temporal variance of fitness over time, thus improving the long-term fitness for the whole population over several generations (Philippi & Seger 1989).

**Fig. 1:**
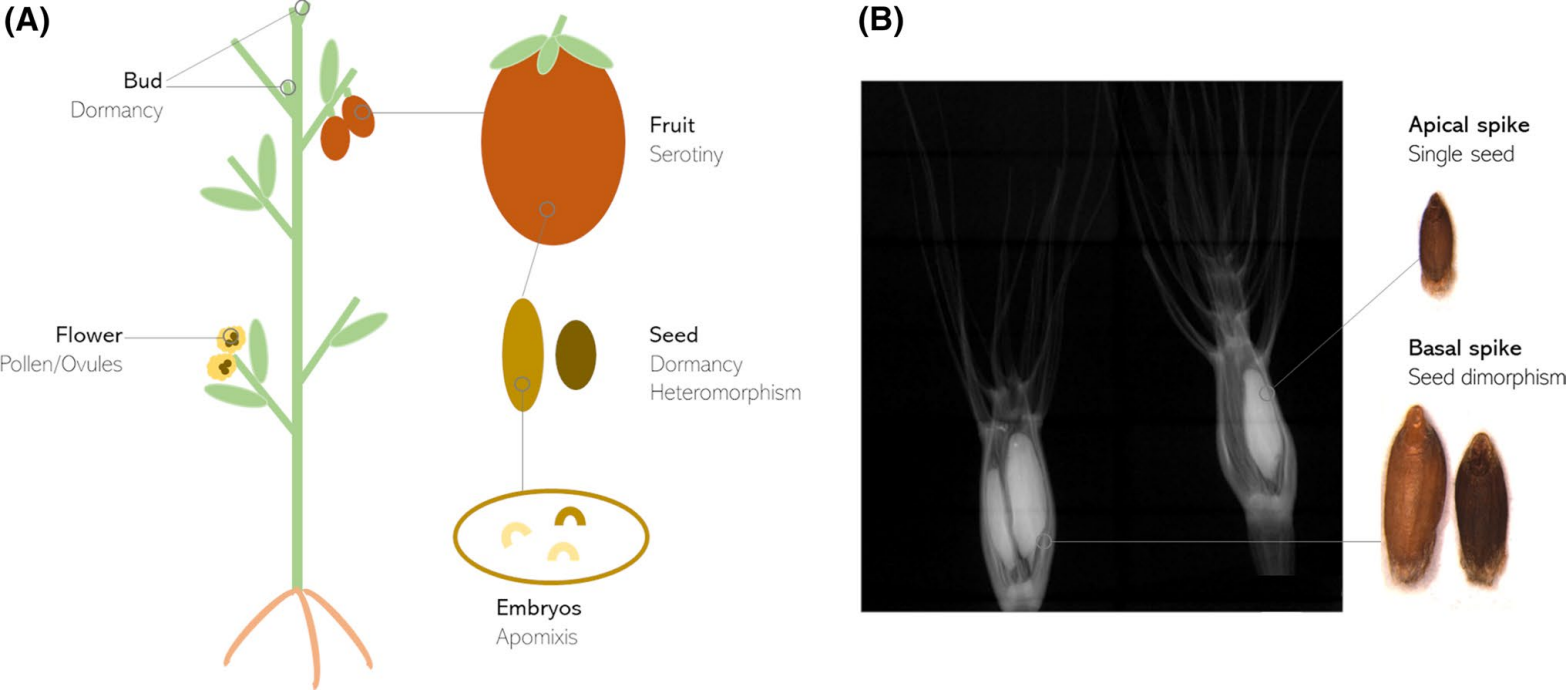
**a** Examples of plant anatomical parts showing bet-hedging strategies, **b** Example of caryopses heteromorphism in Poaceae: *Aegilops geniculata* Roth (photo and X-ray scan: F Guzzon)

Bet-hedging is a widespread strategy in flowering plants and can be observed in different phases of the plant reproductive cycle, mainly at the seed stage but also at the gametic level (pollen/ovules), in the embryos or fruits, and occasionally also in vegetative tissues such as buds (Fig. 1a) (Charlesworth 1989; Cohen 1966; Nilsson et al. 1996; Peters et al. 2011; Thurlby et al. 2012). It is particularly evident in wild plant species adapted to unpredictable environments, in terms of abiotic factors such as rainfall or soil salinity (e.g. ruderal areas or intertidal zones) or biotic components (e.g. host-parasite cycles, predation) (see, e.g. Guzzon et al. 2018; Long et al. 2015; Verin & Tellier 2018; Volis and Bohrer 2013). On the contrary, bet-hedging has been subjected to negative selection during crop domestication in favour of rapid and uniform germination and field establishment even under sub-optimal conditions (Mitchell et al. 2017).

Given the importance of bet-hedging in angiosperms, we review its ecological and evolutionary implications with a focus on seed dormancy and heteromorphism, but also considering special cases such as bud dormancy, apomixis and serotiny. Despite a conspicuous number of studies describing bet-hedging at the ecological level, especially investigating how morphology and germination patterns affect fitness of different plant populations and taxa, still little is known about the molecular mechanisms underlying this phenomenon. We start by providing a resume on the different definitions and categorizations of bet-hedging, moving to its diffusion in flowering plants and its consequences for the dynamics of population genetics. We then review the genetic bases of seed heteromorphism, citing the most recent works linking different loci to bet-hedging strategies. We also describe how transcription and its regulation can drive bet-hedging in plants, including via epigenetic mechanisms triggered by environmental cues. Finally, we provide information on the physiological aspects that are linked to bet-hedging, such as hormonal regulation and antioxidant responses, and we conclude by suggesting further research that is needed on this subject.

### The bet-hedging strategy: definitions and ecology

**Fig. 2:**
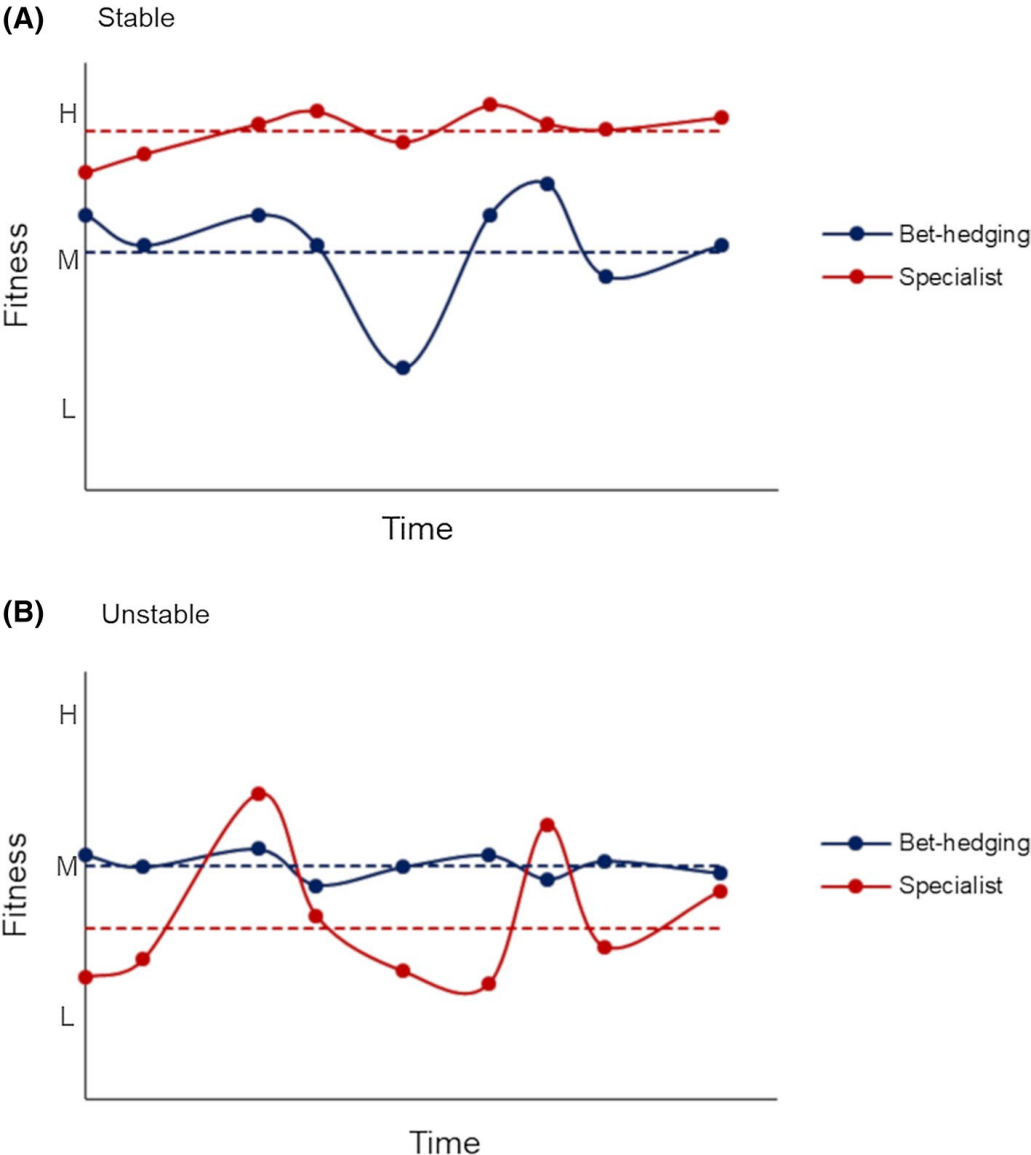
Trends of fitness in bet-hedging and specialization strategies in **a** stable and **b** unstable environments over time L = low; M = medium; H = high fitness. Dashed lines represent the geometric mean of fitness for each strategy. Comparisons are made between strategies, not environments

In a stable environment with predictable fluctuations, the fitness of phenotypes that are specialized to those conditions is maximized and constant over time, and thus, variance of fitness is low or null. In the same environment, generalists are disadvantaged as, even if with low variance, their mean fitness is lower (Fig. 2a). On the contrary, in an unstable environment, specialization can maximize fitness only in the timeframe of particular conditions, while increasing the variance of fitness over time because fitness dramatically decreases when those particular conditions are not met (Olofsson et al. 2009). In such cases, generalist strategies sacrifice the mean fitness in order to lower the variance over time as fitness remains constant over most environmental conditions (Fig. 2b). The so-called ‘bet-hedging’ strategy describes the adoption of a reproductive strategy that maximizes the long-term fitness to ensure survival when coping with an unpredictable environment (Philippi and Seger 1989; Slatkin 1974) or in the presence of fluctuating natural selection (Simons 2009).

In other words, bet-hedging is a trade-off between the mean and the variance of fitness (Philippi and Seger 1989); it results in a reduction in the temporal variance in fitness and a lowered arithmetic mean fitness but in a higher overall (geometric mean) fitness over time under unstable conditions (Fig. 2). Moreover, a strategy that favours diversification within the same generation results in a reduction of competition among siblings, thus uncoupling the survival and fitness of one individual from those of its siblings. Correlation of fitness among individuals is therefore decreased, making bet-hedging a triple trade-off among mean, variance and correlations of fitness (Starrfelt and Kokko 2012).

Olofsson et al. (2009) defined four main types of bet-hedging strategies: (i) *conservative*, or ‘playing safe’: a consistent, low-risk, generalist strategy, e.g. large seeds produced yearly; (ii) *diversified*, several specialized strategies at once but fixed, e.g. variable seed sizes drawn from a fixed distribution; (iii) *‘adaptive coin flipping’*, e.g. randomly produced seed sizes; and (iv) *combined*, any combination of the above.

Diaspore heteromorphism, or the production by the same individual of two or more seed/fruit types, is considered one of the main bet-hedging strategies in flowering plants (see, e.g. Fig. 1b) (Imbert 2002; Venable 1985). Baskin and Baskin (2014) divide seed/fruit heteromorphism in two main categories: heterodiaspory and amphicarpy. Heterodiaspory occurs when ‘two or more types of diaspores are produced above ground and differ in ecological function, e.g. dispersal and dormancy’. Heterocarpic species show different fruit types, with heteroanthrocarpic species possessing segmented fruits bearing different seed morphs and amphi-basicarpic species producing flowers and fruits on both aerial and basal parts of the plant. Heterospermy is another type of heterodiaspory that involves the production of different seed morphs in one or more fruit types. Amphicarpy occurs in plants producing ‘one or more than one type of fruit both above- and below-ground that differ in ecological function’ (Baskin and Baskin 2014).

Venable (1985) described two types of heteromorphism based on the mean fitness and its variance. In the *high risk/high risk* (HRHR) type, two seed morphs are specialized for two different conditions (e.g. wet/dry years), thus maximizing the mean and the variance of fitness of both seeds. On the contrary, in the *high-risk/low-risk* (HRLR) heteromorphic species both seed types are specialized for the same conditions (e.g. wet years), but the fitness of one morph is higher in optimal conditions and lower in different conditions, thus increasing both its mean and variance of fitness (*high risk*) compared to the other morph (*low risk*), whose mean fitness is lower but less variable over time. Seed heteromorphism is common in soil seed banking, in which a fraction of seeds remains dormant after dispersal within the soil or on its surface (Imbert 2002; Venable 2007). A proportion of one morph’s seed cohort remains dormant for a period, influencing fitness in a temporal way by reducing sibling competition, overcrowding and/or inbreeding. In general, the formation of persistent soil seed banks is a bet-hedging strategy that enhances survival and decreases the probability of reproductive failure when the environmental conditions are not favourable (Cohen 1966).

Diaspore heteromorphism can affect fitness also in a spatial manner, for instance, with the endowment of different dispersion capabilities depending on morphology in order to reduce competition. As an example of this, *Picris echioides* L. (Asteraceae) produces two types of achenes: the peripheral ones are dispersed by mammals and remain enclosed in the involucral bract, while the central achenes are wind-dispersed (Imbert 2002). Dispersal itself can be considered a type of diversified bet-hedging, as it can reduce competition among siblings and correlation in fitness between individuals, diversifying their fitness in a spatial manner within the same generation (Hopper et al. 2003; Starrfelt and Kokko 2012). In general, dormancy and dispersal provide bet-hedging strategies to plants experiencing variable environmental conditions in space and time, with the two traits often negatively associated (Buoro and Carlson 2014; de Casas et al. 2015). Indeed, trade-offs in dispersal and dormancy are observed in dimorphic species, where one morph shows a high dispersal (HDi) capability coupled with low or no dormancy levels (LDo), while the other is characterized by low dispersal ability (LDi) and high dormancy (HDo). This is not limited to true dimorphism, as a gradient of strategies following this kind of trade-off was observed along the continuum of diaspores produced from the basal to the aerial parts of the plant by the amphi-basicarpic *Ceratocarpus arenarius* L. (Lu et al. 2013). However, where dormancy and dispersal coevolve, they can become positively associated, so HDo/HDi and LDo/LDi strategies are observed (e.g. in *Aethionema arabicum* (L.) Andrz. ex DC) (Arshad et al. 2019). This species produces two fruit morphs, one being dehiscent and bearing quickly germinating, mucilaginous seeds, and the other fruit being indehiscent with dormant seeds. Mucilaginous seeds germinate quickly after anchoring to the soil near the mother plant, while the fruit with the dormant morph is dispersed for long distances, mainly with rainfall but also by anemochory in the case of hydrothermal stress, due to its winged pericarp (Arshad et al. 2019; Bhattacharya et al. 2019). Moreover, different site-specific offspring ratios are produced, shifting the dimorphic fruits’ migration based on the fluctuations of environmental temperature sensed by the plant, as modelled mathematically by Nichols et al. (2020).

Therefore, heteromorphic systems can adopt within- or between-generations bet-hedging strategies, as diaspores with different behaviours can be produced within the same cohort or in different reproductive seasons. For instance, seed types produced in heteromorphic species can differ in several traits, such as:

- Morphological characteristics: seed mass, colour, hardness of the seed coat (Guzzon et al. 2018; Xu et al. 2016);
- Tolerance to abiotic stresses: salt stress, osmotic stress (Bhatt and Santo 2016; Datta et al. 1970);
- Susceptibility to predation (Hulme 1998);
- Longevity and persistence in the soil seed bank (Guzzon et al. 2018; Zinsmeister et al. 2020);
- Seed dormancy levels (Philippi 1993).

Obviously, several of the aforementioned traits interact in shaping bet-hedging strategies. In wheat wild relatives of the genera *Aegilops* L. and *Triticum* L.(Poaceae), heteromorphic caryopses are produced within the same spike and variations in colour, mass, dormancy, abiotic stress tolerance, longevity and phenology can be observed among the different morphs, contributing to the adaptation to ruderal or disturbed environments (Datta et al. 1970; Guzzon et al. 2018). Similarly, the seed morphs produced by the halophyte *Suaeda salsa* (L.) Pall. (Chenopodiaceae) differ in several traits that allow the adaptation of this species to saline soils, including different dormancy levels, seed coat thickness and colour, and resistance to saline stress (Xu et al. 2016; Zhao et al. 2018). In this species, the variance in seed size in the offspring depends on the interaction between the maternal seed morph and the offspring seed morph. Moreover, the seed types ratio observed in the offspring is influenced by the seed type of the mother plant (Jiang et al. 2019). Recently, also in *Suaeda aralocaspica* (Bunge) Freitag & Schütze variations in seed heteromorphism, in terms of plant size, seed number and heteromorphic seeds ratios, have been associated with maternal environmental factors (Cao et al. 2020).

As pointed out by Long et al. (2015), plants that produce large numbers of seeds per generation should persist better in the soil seed bank, given the fact that a larger number enters the soil and that they tend to be smaller and longer-lived. On the other hand, Huxman et al. (2008) emphasized that high seed production is observed in those species that perform well in favourable seasons but that do not survive in other conditions, e.g. due to low tolerance to drought stress, thus showing high variance in fitness over different years/reproductive seasons. Further research is needed to clarify the possible trade-offs among seed number, persistence and fitness.

Seed sensitivity to environmental factors such as temperature (T) and water potential (*Ψ*) can be modelled to derive the threshold values that permit germination in a fraction of the seed population (Bradford 2018). These models, called ‘population-based thresholds’ (PBT), can be good descriptors for bet-hedging strategies in plant communities as they allow quantitative evaluations of the seed response to environmental fluctuations. For example, higher germination plasticity in a desert community can be achieved when the median value of base water potential, *Ψ*_b_(50), is lower and its standard deviation is higher. Indeed, larger water potential ranges allow larger differences in the germination fraction over different years or rain events within the same year, thus leading to plastic germination and bet-hedging strategies (Liu et al. 2020). Moreover, high variance in year-to-year seed production per each seedling, hence higher variance in fitness, has been associated with small seeds and hydrothermal traits (Ψ and T) linked to slow and fractional germination (Huang et al. 2016). A special case of bet-hedging can be observed in plants that show mixed strategies in terms of embryo production, generating diploid embryos sexually and asexually (i.e. agamospermy or apomixis). Indeed, the co-option of these two mechanisms, which can also lead to the formation of polyembryonic seeds, can provide several advantages. While sexual reproduction leads to gains in genetic diversity due to its intrinsic recombination processes, asexual embryos can assure survival when sexual reproduction is energetically costly and can better preserve genomes that are well adapted to certain environmental conditions (Niklas and Cobb 2017; Thurlby et al. 2012).

Another form of bet-hedging is serotiny, or retention of seeds upon the mother plant at least until the ripening of another seed cohort. Seed expulsion usually happens when the environmental conditions are favourable or in a gradual fashion, e.g. in different seasons, to increase the chances for the seedlings to find an appropriate time window for establishment (Peters et al. 2011). Serotiny is present within the genus *Mammillaria* (Cactaceae): in *M. pectinifera* F.A.C. Weber seeds are expulsed actively during rainy periods and passively over years in order to spread the chances of seedling establishment over time (Peters et al. 2011). The serotinous species *M. hernandezii* Glass and Foster retains a fraction of seeds to protect them from predation and prepares them for dispersal via a rainfall-induced priming process: seed hydration enables a phase of pre-germinative metabolism followed by dehydration that speeds germination and seedling establishment after expulsion from the fruit (Santini and Martorell 2013). In fire-prone environments, ‘weak’ serotiny, i.e. seed retention for < 10 years, is an effective strategy when the interval between two fires exceeds the plant lifespan and there is stochastic variability in fire occurrence. On the other hand, when fires are predictable, the opposite strategies ‘strong’ serotiny (retention > 10 years) and nonserotiny become advantageous specialist adaptations (Lamont et al. 2020).

The production of a diversified population of offspring, in terms of morphs, sizes and number among generations, which is a combination of diversified and adaptive coin-flipping bet-hedging, is theorized as the optimal reproductive strategy for an individual (Olofsson et al. 2009). In this situation, different generations are subjected to different selective pressures, while seeds belonging to same generation experience more or less the same conditions. On the other hand, within-generational bet-hedging is favoured when seeds belonging to the same generation are subjected to different selective pressures, e.g. predation affecting some individuals and not others (Hopper et al. 2003). Indeed, predation affects the more common seed phenotypes more strongly compared to the rare ones (Horst and Venable 2017). Also, predatory pressure can be heavier in certain time windows, for example, some rodents are more active from late spring to autumn. This can favour a within-generational bet-hedging strategy with the production of seeds possessing different dormancy behaviours and therefore a scattered germination pattern during the year (Gremer and Venable 2014). Indeed, assuming that dormancy and within-season germination phenology evolve independently, variance in the latter can favour earlier phenology when dealing with biotic stresses, while later phenology is selected when managing abiotic stresses. Moreover, when modelling within-season mortality and continuous reproduction in these systems, evolutionary branching can arise, with multiple individuals presenting different germination strategies, coexisting but reproducing at different timings (ten Brink et al. 2020).

Bud dormancy enables the repeated phase of rest that punctuates periods of growth in the life cycle of many perennial species (Cooke et al. 2012). It has also been proposed to be a bet-hedging strategy in response to herbivory predation, if it results in lower seeds production in years of low herbivore pressure and reduces the variance of seed production in time with compensatory effects in years of intense pressure (Nilsson et al. 1996).

Moreover, also among different populations of the same species there can be variation of seed production depending on the environment experienced by the population, thus again depending on the selective pressure(s) exerted by the environment (Dyer 2017; Philippi 1993). However, Starrfelt & Kokko (2012) suggest that bet-hedging is a continuum of strategies rather than divided into distinct categories, in terms of degree of conservation/diversification among different populations of the same taxa and also within/among generations. This latter model has been demonstrated mathematically by Haaland et al. (2020), also predicting a continuum in terms of among‐ versus within‐individual phenotypic variation affected by the amount of environmental stochasticity experienced and tuned in order to maximize the genotype fitness.

### Phylogenesis and distribution in the Angiosperms

**Fig. 3:**
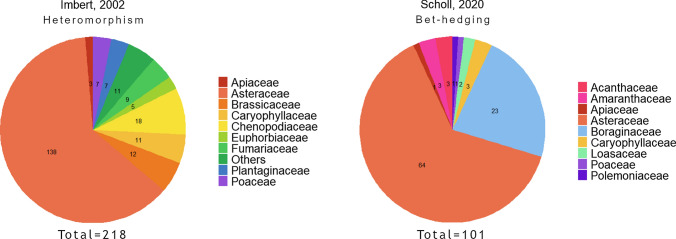
Number of heteromorphic species and of species showing bet-hedging strategies distributed in different plant families as reported by Imbert (2002) and Scholl et al (2020), respectively.

Bet-hedging strategies are diffused in many plant families in different plant development phases and anatomical parts, but to the best of our knowledge, it has been phylogenetically quantified only in terms of seed heteromorphism and persistence within the soil seed bank. Lamont and colleagues (2020) describe the phylogenetic distribution of serotiny, present in eight angiosperm families; however, this distribution is not yet resolved at the species level. Heteromorphism was reported to be present in 218 plant species and is more frequent in dicotyledonous plants (16 families out of 18 are dicots; Imbert 2002) (Fig. 3). Wang and colleagues in 2010 listed additional heteromorphic species, raising the total count to 292. Recent work by Scholl and colleagues (2020) examined the presence of seed heteromorphism in 101 angiosperm species, distributed across 51 genera and 9 families, mainly within Asteraceae and Boraginaceae (Fig. 3). This analysis is the first to directly link heteromorphism to bet-hedging strategies at the phylogenetic level by considering the association with different factors: aridity, coefficient of variation (CV) of precipitation, life-span (annual/perennial) and weediness of the species. A significant correlation was found between heteromorphism and aridity, while weediness and annual life cycle were not significant predictors, in contrast with previous hypotheses (Imbert 2002; Scholl et al. 2020). Even if the resolution of the phylogenetic analysis could be biased by the occurrence locations of the considered taxa, as the authors predominantly considered North America, this paper gives insight on the diffusion of seed heteromorphism as a bet-hedging strategy in the Angiosperms.

Recently, another phylogenetic analysis highlighted the presence of soil seed banking in more than 2500 angiosperm taxa having different life cycles (annual/perennial, weedy/woody), habitats and seed traits such as dormancy. Persistent seed banks, suggesting bet-hedging strategies, are mostly diffused in weedy taxa with annual life cycles living in disturbed and ruderal habitats (Gioria et al. 2020). Given the lack of association with life cycle and weediness found by Scholl et al. (2020) in heteromorphic bet-hedgers, a comprehensive phylogenetic analysis that considers both persistence of the seed bank and heteromorphism is needed.

Diaspore heteromorphism, while observed in crop wild relatives (e.g. wheat wild relatives: Gianella et al. 2020; oat wild relatives*, Avena sterilis* L.: Volis 2014; wild *Polygonum erectum* L.: Mueller 2017; lupin wild relatives, *Lupinus angustifolius* L.: Moncalvillo et al. 2019), is often lost in crops as a result of selection for uniform and quick germination as part of the domestication process. Indeed, the traits that characterize the ‘domestication syndrome’, i.e. plant traits that mark the divergence of domesticated crops from their wild progenitors, are often reproductive traits that affect yield, such as seed size and number or shattering at maturity (e.g. wheat) (Iriondo et al. 2018; Nave et al. 2016). In agricultural contexts, bet-hedging strategies are indeed disadvantageous as they result in staggered germination and seedling establishment, reducing yield and complicating pest and growth management (Mitchell et al. 2017). Hence, comprehensive analyses of the genetic bases of bet-hedging strategies are of great importance, in particular in genetic drift studies about crop domestication by comparing presence/absence of this trait in crops and their wild relatives.

### Population dynamics

Long-term soil seed banking connected with bet-hedging strategies can modify the dynamics of population genetics in different ways. These include (Tellier 2018): (i) reducing the extinction rate in unpredictable environments, thereby influencing population size and genetic drift; (ii) through persistence in the soil, lengthening the time to the point where two lineages coalesce in their most recent common ancestor (MRCA); (iii) increasing the genetic recombination rate since the coalescent time is lengthened, leading to higher genetic diversity and lower linkage disequilibrium; (iv) potentially increasing the mutation rate due to the time spent in the soil; (v) reducing both the fixation rate of favourable alleles and the risk of allele loss by random drift, thereby affecting the rates and signatures of natural selection; and (vi) reducing inbreeding.

In particular, it has been demonstrated that long-term soil seed banking seeds show an equal or higher nucleotide substitution rate when compared to less persistent seeds, and that the majority of seed bank-borne mutations are neutral or nearly neutral, in accordance with Otha and Kimura’s neutral theory of molecular evolution (Dann et al. 2017). Moreover, differences in substitution rates can be observed at the interspecific level but also intra-taxa at the same locus, depending on specific population traits such as generation time or altitude and latitude (Dann et al. 2017).

The evolution and diffusion of soil seed banking in different taxa and populations is not only linked to unpredictable climatic conditions but also to unstable co-evolutionary dynamics between host and parasites: seed banking is evolutionarily favoured when the cost of alleles for resistance to parasites and the disease severity are high (Verin and Tellier 2018). This is more likely to happen in more stable environments (e.g. temperate areas), where infections are favoured and host-parasite cycles can be chaotic, making the host’s biotic environment unstable (Verin and Tellier 2018). Predation is an evolutionary driving force for the constitution of persistent seed banks; for instance, in grasslands the predatory pressure exerted by rodents favours the establishment of seed banks in grasses and legumes (Hulme 1998). This selective force acts not only on the degree of permanence within the soil but also on seed size, existing as a negative correlation between these two seed traits (i.e. smaller seeds persist more in the soil seed bank) (Volis & Bohrer 2013). Indeed, larger seeds are predated preferentially in both pre- and post-dispersal stages and represent a higher metabolic cost when remaining dormant in the soil compared to small seeds. These two features constrain larger seeds to evolve, on average, a quicker germination strategy and transient seed banks (Hulme 1998).

### Genetic bases of bet-hedging

Although the adaptive significance of bet-hedging in seeds and fruits has been studied quite extensively from the point of view of morpho-ecology, little is known about its basis at the molecular level.

Some early studies focused on macro-differences in molecular features of heteromorphic species. For example, karyotypic variations in terms of chromosomal morphology and length, together with differences in electrophoretic profiles of total seed proteins, have been linked to different morphs in the achenes of *Calendula micrantha* Tineo & Guss. (Asteraceae) (Soliman 2003). The morphologic variance of distinct seed sets of *Primula vulgaris* Hudson (Primulaceae) was explained, in part, by genes linked to flower morphology and the influence of the light and temperature environment experienced by the female parent during the flowering period (Vaerbak & Andersen 2004). Another approach used was the utilization of amplified fragment length polymorphisms (AFLP) in *Packera tomentosa* (Asteraceae) to genetically differentiate clones (genets) that showed cryptic heteromorphism, i.e. a variable seed behaviour such as dormancy that is not accompanied by a discriminant morphological variation (Imbert 2002). While differences in morphology were not evident, seed mass and germination performance differed among genets and seed positions within the flowers (Leverett and Jolls 2013).

More recently, Nave and colleagues (2016) analysed the molecular bases of within-spikelet heteromorphic caryopses exploiting a recombinant inbred line (RIL) population originated from durum wheat (*Triticum turgidum* subsp. *durum* (Desf.) Husn.) and its progenitor, wild emmer wheat (*Triticum turgidum* subsp. *dicoccoides* (Asch. & Graebn.) Thell.) (Poaceae), in order to characterize heteromorphism to clarify the domestication syndrome in wheat. While domesticated emmer shows uniform grain size and germination, wild emmer bears, within the same spikelet, bigger caryopses located in the upper part that germinate more rapidly when compared to their smaller siblings located in the lower part of the spikelet. A quantitative trait locus (QTL) for uniform grain size and germination on chromosome 4B (*QGD-4BL*) explained a high proportion of within-spikelet variation in terms of caryopses dimensions and dormancy, indicating a modification selected during early stages of domestication. (Nave et al. 2016).

On chromosomes 3 and 5 of *Arabidopsis thaliana* L. (Brassicaceae), two loci, one overlapping with *DELAY OF GERMINATION 6 (DOG6)* and the other with *DOG1* (two of the loci underlying the extent of dormancy under different environmental conditions; Bentsink et al. 2010) have been associated with a bet-hedging strategy in seeds belonging to the same siliques when coping with environmental stresses. Seeds subjected to short strong stresses, e.g. heat shock at 49 °C for 30 min, showed differential germination timing that allows part of the seed cohort to survive an unpredictable period of unfavourable environmental conditions (Abley et al. 2020). Also, two proteins encoded by *FLOWERING LOCUS C (FLC)* and *FLOWERING LOCUS T (FT)* enable *A. thaliana* mother plants to modulate seed dormancy in the progeny depending on the external temperature, sensed through the epigenetic state of these genes. This mechanism allows the modification of dormancy levels through the regulation of seed coat development and hormone production in response to environmental conditions during seed maturation to diversify the behaviour of the progeny, presumably to maximize fitness in the following generation (Chen and Penfield 2018; Springthorpe and Penfield 2015). Sensitivity to environmental signals for dormancy release also occurs when other key germination-repressing genes are expressed at low levels (e.g. *DOG1*, *CIPK23*—*CBL-interacting serine/threonine-protein kinase 23*, *PHYA—Phytochrome A*), and subtle differences in the seed response contribute to bet-hedging via the formation of transient or persistent seed banks (Footitt et al. 2014). The formation of seed banks in *A. thaliana* can be linked to the differential response to chilling in terms of primary dormancy release, with mixed autumn- and spring-germinating cohorts observed within populations in the same year. This system has been subjected to genetic and molecular analyses that support a role for *DOG1* in determining the depth of dormancy, but identified other loci more closely associated with dormancy cycling (Footitt et al. 2019). Recent work by Martínez-Berdeja and colleagues (2020) linked primary dormancy release induced by chilling in *A. thaliana* seeds to *DOG1* haplotype identities through a genome-wide association study.

Two populations of *Brassica oleracea* L. (Brassicaceae), genetically identical except for two loci involved in abscisic acid (ABA) catabolism (*RABA1—Reduced ABA 1*) and sensitivity (*SOG1—Suppressor of gamma response 1*), showed a bet-hedging strategy based on a continuum of ABA-dependent dormancy. Allelic differences at these loci were correlated with dormancy release when seeds were subjected to different temperatures, with the two lines showing different lower limits of tolerance (Awan et al. 2018).

A special case of bet-hedging is exhibited by *Syzygium paniculatum* Gaertn (Myrtaceae). This rare tree species relies on a mixed reproductive strategy utilizing both sexual embryonic production and agamospermy(Thurlby et al. 2012). Seeds are polyembryonic, and when dimorphic, the sexual embryo is bigger than the asexual one, whereas, when polymorphic, the embryos are the same size, apparently due to the increased competition from multiple asexual siblings. Overall, genetic diversity in this species is low, as observed when different populations were compared using nuclear simple sequence repeat (nSSR) markers. This mixed reproductive strategy can be seen as a form of bet-hedging, as the sexual embryos represent an adaptive advantage for maintaining genetic diversity, while asexual embryos assure dispersion and survival even when sexual reproduction fails or is too costly (Thurlby et al. 2012).

Although there is some evidence for single dominant loci being responsible for apomeiosis and parthenogenesis in some grasses, several genes have been associated with apomixis in Poaceae: *ASGR-BABY BOOM-like (PsASGR-BBML)* gene from *Pennisetum squamulatum* (L.) R.Br. and *BABY BOOM1* (*BBM1*) in rice (*Oryza sativa* L.) (Conner et al. 2015; Khanday et al. 2019). Khanday and colleagues (2019) demonstrated that the ectopic expression of *BBM1* in egg cells of rice is sufficient for parthenogenesis but that fertilization is still required for seed endosperm production; notably, this system shows the feasibility of clonal propagation through apomictic seeds in crops. In addition to a genetic basis, epigenetic regulation of apomixis has also been hypothesized, since the latter could explain the facultative nature and reversibility to sexual seed production (Kumar 2017; Rodrigues and Koltunow 2005).

### Bet-hedging and non-genetic bases: transcription

Non-genetic variation could play a major role in terms of phenotypic variation in the context of bet-hedging. Selection for diversification often implies low heritability of fixed genetic adaptations because the latter is the ratio of additive genetic variance to the total phenotypic variance (Simons and Johnston 2006). On the other hand, a major source of molecular variation is differential gene expression and its regulation. Transcriptional variability (often referred to as ‘transcriptional noise’) can be caused by environmental fluctuation or other constraints (e.g. at the cellular level by physical position in a cell population, differential cell functions) and can result in detrimental or beneficial phenotypic variability (Mitchell et al. 2017). Wide variation in transcript levels, when interpreted as noise, suggests poor regulatory control or unavoidable stochastic ‘error’. The development of technologies to assess transcriptomes at the single-cell level has provided different interpretations of what has been designated as transcriptional ‘noise’ (Stadler and Eisen 2017). Pooled samples inevitably bulk together the transcripts from populations of individuals, whether of seeds or cells. However, biological variation can be interpreted in terms of populations of individuals in which response thresholds and physiological/transcriptional activities vary in reproducible or programmed ways. Thus, transcriptional variability may represent differences among cells in a population that is part of the regulatory process, rather than representing uncontrollable error. In fact, population models based on threshold-dependent dynamics have successfully described diverse aspects of seed germination behaviour, including responses to temperature, water potential, hormones, dormancy, ageing, respiration rates and other parameters (Bradford 2018). Similarly, recent single-cell in vivo transcriptional studies revealed that ‘plant tissues respond to external signals by modulating the number of cells engaged in transcription rather than the transcription rate of active cells’ (Alamos et al. 2020). An important implication of this is that experimental designs for examining seed molecular biology will need to move toward analyses of individual seeds (e.g. Still and Bradford 1997) rather than pooling multiple seeds per sample, which inevitably combines seeds in different physiological or developmental states, e.g. dormant and non-dormant or germinating vs non-germinating, at a given time.

It is evident that seed-to-seed variation is a fundamental component of bet-hedging strategies. For instance, beneficial variability can be observed in the halophyte *S. salsa* which shows differential transcription to adapt to environmental fluctuations in soil salinity (Xu et al. 2017). This species produces two seed morphs: brown seeds germinate faster and tolerate higher salt concentrations, while black seeds remain dormant, tolerate lower salt concentrations and constitute the soil seed bank. The transcriptomic profiling performed by Xu and colleagues (2017) on mature dry seeds revealed that the two morphs show differential expression of 4648 genes, mainly belonging to pathways related to embryo development, fatty acids metabolism, osmotic equilibrium and hormonal regulation, indicating their different developmental trajectories. In particular, genes involved in the metabolism of two organic osmolytes, betaine and proline, were upregulated, the latter being not only involved in osmotic regulation but also in the prevention of germination in stressful conditions by maintaining the embryo axis in a resting state.

In *Ae. arabicum* (Brassicaceae), fruit dimorphism (dehiscent or indehiscent) is a ‘last-minute’ developmental decision, happening after fertilization. In this species, flowers that already possess the structures typical of the dehiscent morph (four to six ovules and a segment) can degrade those structures and instead form indehiscent fruits (Lenser et al. 2018). This bet-hedging strategy allows plastic responses to environmental unpredictability in the current season. Indeed, the ratio of single-seeded indehiscent fruits, bearing dormant seeds, to multi-seeded dehiscent fruits, bearing quickly and uniformly germinating seeds, increases in adverse growth conditions such as defoliation or shading. Lenser and colleagues (2018) demonstrated that the production of these two fruit morphs is regulated by the transcript levels of *BRANCHED1 (BRC1)*, belonging to the family of transcription factors *TB1 CYCLOIDEA PCF (TCP)*. *BRC1* transcription is indeed particularly important in hormonal production and is thought to integrate the regulatory roles of developmental hormones such as auxin, strigonolactone and cytokinin that are involved in fruit development (Lenser et al. 2018). Moreover, a transcriptome analysis revealed differentially expressed genes (DEGs) in dry seeds belonging to the two fruit morphs. The ‘less dormant’ morph showed higher levels of transcripts of genes whose expression increases with maturation, such as genes encoding ribosomal proteins, LEAs, NYE1 protein (chlorophyll degradation) and HSFA9 (heat shock protein), and lower expression of genes that decrease during maturation such as *ABI3* (abscisic acid-mediated regulation and embryo degreening) and *WRI1* (embryo development). These results, suggesting different degrees of after-ripening in the two morphs, are in accordance with the already known dormancy behaviour of the two morphs, while other DEGs (related to ROS detoxification, late embryo abundant proteins-LEAs) suggest also the possibility of different longevity behaviour, so far unknown (Wilhelmsson et al. 2019).

### Bet-hedging and non-genetic bases: epigenetics

Changes in environmental cues can modify gene expression through epigenetic mechanisms (i.e. methylation, histone modifications and non-coding RNAs-mediated), generating plastic phenotypic variation that can be heritable or not. Phenotypic plasticity can thus be individual (within-generation) or be inherited as an effect of the epigenetic changes exerted by the environment on the parents that is meiotically transmitted (trans-generational). The latter can be defined as ‘heritable bet-hedging’ and can maintain or increase the genetic potential of a population across generations, acting as a means of evolutionary rescue from extinction (Herman et al. 2013; O’dea et al. 2016). Epigenetic stability across several generations can be seen as a trade-off between short-term, within-generation epigenetic changes and long-term, genetically fixed adaptations (Alvarez et al. 2020). Random switching between epigenetic states is indeed advantageous in situations of high unpredictability, where there is not an optimal phenotype adapted to all the possible environmental conditions, while genetically fixed adaptations are advantageous for phenotypes close to the optimum, in less variable environments. When intermediate situations occur, i.e. variations in the environment exist but are not so frequent, trans-generational heritability of randomly generated epiallelic variants is advantageous as an evolutionary strategy that maintains adaptive phenotype-environment matching at an intermediate temporal scale (Herman et al. 2013). Thus, persistent trans-generational effects become adaptive when a response to long-term, multigenerational environmental changes is necessary. In this context, ‘fixed’ epigenetic states are not directly inherited as epigenetic signatures, rather they are re-induced through a feedback between phenotype and the environmental cues/conditions (Alvarez et al. 2020). The proportion of variation in seed traits related to dispersal that cannot be explained by genetic or environment variability and that can be transmitted to subsequent generations has its origins in epigenetics, which can be both heritable and adaptive (Johnson et al. 2019). The heritability of epigenetic marks was explored in four different *A. thaliana* inbred lines after a heat stress induced in an ‘ancestral’ generation and then studied in the third generation. One genotype (Cvi) showed the establishment of a bet-hedging strategy possibly induced by the inheritance, within the same generation, of random epiallelic variants caused by the heat stress experienced by the ancestor (Suter & Widmer 2013). In another study on four *A. thaliana* lines, no transgenerational epigenetic heritability was found when plants were subjected to water stress. Rather, the differences in responses to drought stress observed between generations were based on phenotypic plasticity rather than maternal effects and were exerted in a within-generational fashion (van Dooren et al. 2020).

Epigenetically induced variability can be advantageous in coping with environmental changes, but it can also be maladaptive when random epialleles are generated. Nevertheless, maladaptive epigenetic marks are often negatively selected at the seedling stage, and therefore they result in more tolerance in plants that produce large seed numbers, where the probability of extinction caused by maladaptive marks in the offspring is lower (Minow & Colasanti 2020). So, even with the possible insurgence of maladaptive marks, epigenetically variable populations can be advantageous, as a fraction of individuals is likely to be more suited to certain environmental conditions, thus providing buffering capacity to the total population (Alonso et al. 2018).

Cytosine methylation is a key component of epigenetic regulation in plants and it has been associated with functional changes in gene expression and genomic stability (Alonso et al. 2014, 2018). Genome-wide variations in cytosine methylation are associated with fecundity-related traits in the evergreen shrub *Lavandula latifolia* Medik (Lamiaceae) and in the perennial herb *Helleborus foetidus* Moench (Ranunculaceae) (Alonso et al. 2018; Herrera et al. 2014). In *L. latifolia*, sub-individual epigenetic mosaicism, in which different parts of the same genetic individual differ in DNA methylation patterns, was hypothesized to be related to variations in the exposure of different plant anatomical parts to one or more environmental factors that trigger epigenetic changes as a plastic response, resulting in the differential production of seeds in terms of mass and number (Alonso et al. 2018). In *H. foetidus*, variation in the individual transgenerational transmission of epigenetic marks (mainly methylation) was related to within-plant variance in seed size (Johnston & Bassel 2018), suggesting a complex mechanism, not only related to mosaicism but also to transmissibility that could link epigenetic changes to sub-individual heterogeneity of reproductive organs.

Rapid changes in the dormancy status of *A. thaliana* seeds in response to environmental fluctuations, especially temperature, can also be driven by epigenetic modifications. Genome-wide chromatin remodelling induces changes in gene expression that enable seeds to respond to seasonal variation through different dormancy behaviours, in particular via histone modification of the *DOG1* chromatin with the H3K4me3 and H3K27me3 marks that cause a reduction of DOG1 protein production in late spring and therefore dormancy release (Footitt et al. 2015).

While full details remain to be uncovered, it is clear that inducible changes in genetic networks can increase the range of possible molecular interactions, thereby allowing the expression of plasticity required for the adaptation to biotic or environmental stochasticity.

### Impact on seed physiology

A positive-feedback regulatory motif involved in ABA synthesis and degradation was modelled in relation to germination patterns in *A. thaliana* (Abley et al. 2020; Johnston and Bassel 2018). This motif belongs to a larger regulatory network that involves also gibberellin (GA), which interacts antagonistically with ABA in regulating germination and dormancy (Topham et al. 2017); this system can be tuned in response to environmental cues and provides regulation of transcriptional factors that control germination and dormancy as an adaptation to stressful conditions (Abley et al. 2020). Indeed, a type of bet-hedging strategy observed in seeds of *A. thaliana* subjected to stress has been recently associated with loci overlapping with several genes involved in the regulation of GA and ABA sensitivity/degradation. This regulation, of ABA levels in particular, in turn generates downstream transcriptional variation possibly involved in phenotypic plasticity. Moreover, the authors ruled out a previously hypothesized positional regulatory gradient in the ovary, thus uncoupling a possible developmental influence on dormancy as a confirmation of the effective presence of bet-hedging (Abley et al. 2020).

Differences in physiological responses to oxidative stress have also been observed in heteromorphic species with bet-hedging strategies. In *Arthrocnemum macrostachyum* (Moric.) K.Koch and *Arthrocnemum indicum* (Willd.) Moq. (Chenopodiaceae), two halophytes with black/brown and large/small heteromorphic seeds, respectively, differential levels of antioxidant activity and oxidation markers (H_2_O_2_ and malondialdehyde-MDA) were found during seed germination under increasing salinity. In both species, the salt-tolerant morphs did not show changes in their antioxidant activity nor in H_2_O_2_ and MDA levels, while the morphs with lower salt tolerance also showed a less resilient antioxidant machinery, resulting in higher levels of oxidative damage (Nisar et al. 2019). Similarly, when subjected to accelerated ageing, heteromorphic caryopses of *Aegilops* and of *Triticum urartu* Thum. ex Gandilyan (Poaceae) showed different lifespans and antioxidant activities: the shorter-lived, larger morphs possess lower antioxidant activities when compared to their smaller, darker and longer-lived counterparts (Gianella et al. 2020).

In both studies, darker morphs showed higher phenolic contents and delayed germination. Some polyphenols indeed act as germination inhibitors and slow down water uptake by thickening the seed coat, thus allowing a longer persistence in the soil. For instance, higher phenolic contents in black seeds of *S. salsa* have been associated with a population-dependent bet-hedging strategy linked to persistence in intertidal soils subjected to waterlogging, and also to longevity (Xu et al. 2016). Brown seeds in this species, with lower phenolic contents in their seed coats, germinate quickly due to more rapid water uptake and showed higher contents of free sugars and enzymes related to lipid and pre-germinative metabolism (Xu et al. 2016; Zhao et al. 2018). Similarly, *Atriplex centralasiatica* Iljin (Amaranthaceae) possesses two morphs, black and brown, in which there is differential accumulation of phenolics in the seed coat. The black morph, with slower water uptake, constitutes the soil seed bank, whereas the brown one is salt tolerant and shows a more rapid germination, due to its permeable coat and a higher GA content (Li et al. 2011).

It has been postulated that seeds with deeper dormancy could be less damaged by reactive oxygen species (ROS) due to the ‘collateral’ antioxidant activity of chemicals involved in dormancy regulation, e.g. phenols (Lepiniec et al. 2006). Flavonoids, lignins and lignans found in the seed coat are also associated with seed dormancy and longevity (Long et al. 2015), and polyphenols in general act as protective chemicals fundamental for the persistence in the soil, being antioxidant and also antimicrobial compounds (Hradilová et al. 2019). Differential levels of proanthocyanidins (PAs), a class of polyphenols, were observed in the seed coats of differently pigmented populations of wild pea (*Pisum sativum* subsp. *elatius* (M.Bieb.) Asch. & Graebn. (Fabaceae)) collected across the Mediterranean area, south-eastern Europe and the Middle East. The soluble to insoluble PAs ratio and coat thickness correlated with different dormancy levels in differently distributed populations, this in turn correlating with different seasonality and climatic conditions and thus indicating a bet-hedging strategy in wild peas (Hradilová et al. 2019). An association between seed coat properties and bet-hedging was found also in *Medicago truncatula* Gaertn (Fabaceae), showing plasticity in dormancy release along with an aridity gradient. A genome-wide association study revealed that four genes related to flavonoid metabolism and seven peroxidases and thio-/peroxiredoxins are associated with differential dormancy release depending on the environmental conditions (Renzi et al. 2020).

### Conclusions and future perspectives

**Fig. 4:**
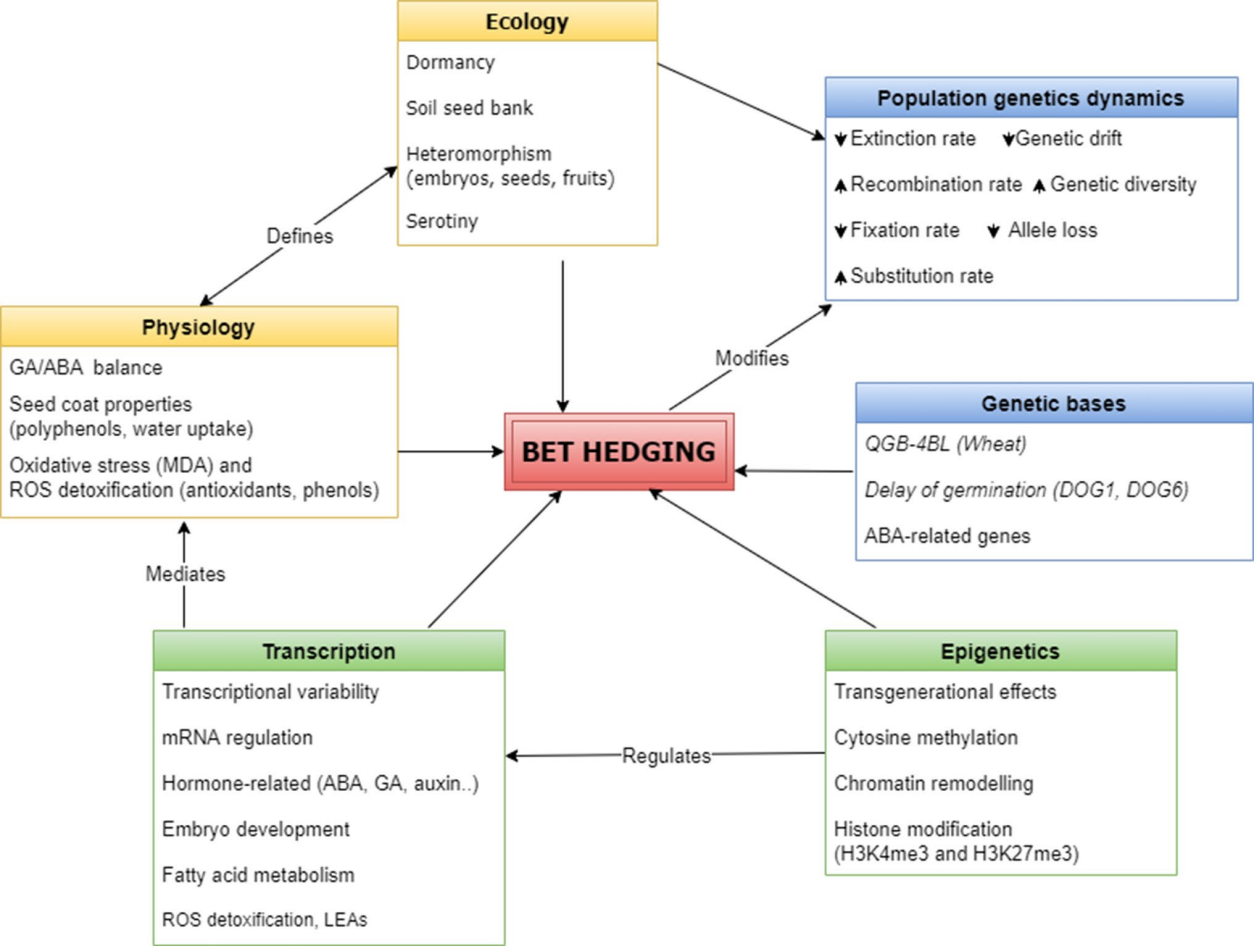
Network of processes related to bet-hedging

Bet-hedging strategies result from ecological adaptions and are driven by different genetic, epigenetic and physiological processes that in turn modify the dynamics of population genetics (Fig. 4):

- Bet-hedging strategies, diffused in probably hundreds of angiosperm species, can be categorized depending on the degree of specialization or risk taken. Different plant anatomical parts (e.g. seeds, fruits and buds) can embody these strategies by exhibiting physical (e.g. seed size) or physiological (e.g. seed dormancy) heteromorphism (Olofsson et al. 2009; Scholl et al. 2020).
- Several traits are influenced by bet-hedging strategies, including abiotic and biotic stress resistance, germination phenology, susceptibility to predation, seed dormancy, seed morphology and seed longevity (Bhatt and Santo 2016; Datta et al. 1970; Guzzon et al. 2018).
- The presence of bet-hedging alters the dynamics of population genetics, in particular modifying the substitution rates and influencing also host-parasite coevolution (Dann et al. 2017; Verin and Tellier, 2018).
- Different loci have been associated with seed heteromorphism and bet-hedging, mainly comprising genes involved in pathways linked to dormancy (e.g. *DOG1* and ABA-related genes). Bet-hedging is also reflected in differential transcription patterns of genes belonging to different metabolic and developmental processes, including embryo development, fatty acids and sugar metabolism, ROS detoxification and late embryogenesis abundant proteins-LEAs (Footitt et al. 2014; Nave et al. 2016; Wilhelmsson et al. 2019; Zinsmeister et al. 2020).
- Epigenetics can drive bet-hedging via trans- and inter-generational transmission by regulating gene expression through genome-wide methylation marks in response to environmental cues. The regulation of different pathways affects physiology through differential hormone levels, antioxidant responses and seed coat properties in heteromorphic seeds (Abley et al. 2020; Alonso et al. 2018; Hradilová et al. 2019).

Many aspects of bet-hedging in flowering plants still need to be explored. From an ecological point of view, most of the studies on bet-hedging have focused on just one phenotypic trait, such as seed longevity (Guzzon et al. 2018) or resistance to abiotic stresses during germination (Bhatt and Santo 2016). Further studies are needed to consider simultaneously the different traits influenced by bet-hedging in order to clarify the ecological trade-offs involved in this strategy in different plant species (Gianella et al. 2020). Moreover, a more comprehensive phylogenetic analysis is needed to assess the presence of bet-hedging and its evolutionary significance in the whole phylum, considering taxa from all over the globe (Scholl et al. 2020; Gioria et al. 2020). Additionally, the genetic bases of bet-hedging are poorly known, particularly in those plant families where it is less distributed. Indeed, studies that could identify the loci underlying bet-hedging strategies in different taxa are necessary, such as studying loci negatively selected for diaspore and germination uniformity during domestication, as elegantly done by Nave and colleagues in durum wheat (2016). Given the important role of non-genetic mechanisms in the plant responses to environmental cues, a focus on epigenetics is also necessary and could complement multi-generational germination studies where seeds are subjected to different stresses resembling the environmental variations that are suspected to be the trigger of the evolution of bet-hedging. Since differential gene expression and different patterns of metabolites have already been observed in some taxa showing bet-hedging strategies (e.g. Nisar et al. 2019; Xu et al. 2017), multi-omics approaches could shed light on the dynamics of pre-germinative metabolism in heteromorphic seeds that show bet-hedging strategies. Indeed, with the recent advancements and the increasing cost-effectiveness of molecular and physiological assays, bet-hedging could be explored at different levels of detail also in non-model organisms, in order to obtain a clearer picture of this fascinating evolutionary strategy.

## References

[CR1] Abley K, Formosa-Jordan P, Tavares H, Chan E, Leyser O (2020). Locke JCW (2020) An ABA-GA bistable switch can account for natural variation in the variability of *Arabidopsis* seed germination time. BioRxiv.

[CR2] Alamos S, Reimer A, Niyogi KK (2020). Garcia HG (2020) Quantitative imaging of RNA polymerase II activity in plants reveals the single-cell basis of tissue-wide transcriptional dynamics. BioRxiv.

[CR3] Alonso C, Perez R, Bazaga P, Medrano M, Herrera CM (2014). Individual variation in size and fecundity is correlated with differences in global DNA cytosine methylation in the perennial herb *Helleborus foetidus* (Ranunculaceae). Am J Bot.

[CR4] Alonso C, Pérez R, Bazaga P, Medrano M, Herrera CM (2018). Within-plant variation in seed size and inflorescence fecundity is associated with epigenetic mosaicism in the shrub *Lavandula latifolia* (Lamiaceae). Ann Bot.

[CR5] Alvarez M, Bleich A, Donohue K (2020). Genotypic variation in the persistence of transgenerational responses to seasonal cues. Evolution.

[CR6] Arshad W, Sperber K, Steinbrecher T, Nichols B, Jansen VAA, Leubner-Metzger G, Mummenhoff K (2019). Dispersal biophysics and adaptive significance of dimorphic diaspores in the annual *Aethionema arabicum* (Brassicaceae). New Phytol.

[CR7] Awan S, Footitt S, Finch-Savage WE (2018). Interaction of maternal environment and allelic differences in seed vigour genes determines seed performance in *Brassica oleracea*. Plant J.

[CR8] Baskin CC, Baskin JM (2014). Seeds-Ecology, biogeography, and evolution of dormancy and germination.

[CR9] Bentsink L, Hanson J, Hanhart CJ, Blankestijn-de Vries H, Coltrane C, Keizer P, El-Lithy M, Alonso-Blanco C, de Andres MT, Reymond M, van Eeuwijk F, Smeekens S, Koornneef M (2010). Natural variation for seed dormancy in Arabidopsis is regulated by additive genetic and molecular pathways. Proc Natl Acad Sci USA.

[CR10] Bewley JD, Bradford KJ, Nonogaki HWM (2013) Seeds: Physiology of Development, Germination and Dormancy, 3rd Edition. Springer 10.15713/ins.mmj.3

[CR11] Bhatt A, Santo A (2016). Germination and recovery of heteromorphic seeds of *Atriplex canescens* (Amaranthaceae) under increasing salinity. Plant Ecol.

[CR12] Bhattacharya S, Sperber K, Ozudogru B, Leubner-Metzger G, Mummenhoff K (2019). Naturally-primed life strategy plasticity of dimorphic *Aethionema arabicum* facilitates optimal habitat colonization. Sci Rep.

[CR13] Bradford KJ (2018). Interpreting biological variation: seeds, populations and sensitivity thresholds. Seed Sci Res.

[CR14] Buoro M, Carlson SM (2014). Life-history syndromes: integrating dispersal through space and time. Ecol Lett.

[CR15] Cao L, Chen L, Wang J, Xing J, Lv X, Maimaitijiang T, Lan H (2020). Effects of genetic and environmental factors on variations of seed heteromorphism in *Suaeda aralocaspica*. AoB PLANTS.

[CR16] Charlesworth D (1989). Evolution of low female fertility in plants: Pollen limitation, resource allocation and genetic load. Trends Ecol Evol.

[CR17] Chen M, Penfield S (2018). Feedback regulation of *COOLAIR* expression controls seed dormancy and flowering time. Science.

[CR18] Childs DZ, Metcalf CJE, Rees M (2010). Evolutionary bet-hedging in the real world: empirical evidence and challenges revealed by plants. Proc R Soc Lond [Biol].

[CR19] Cohen D (1966). Optimizing reproduction in a randomly varying environment. J Theor Biol.

[CR20] Conner JA, Mookkan M, Huo H, Chae K, Ozias-Akins P (2015). A parthenogenesis gene of apomict origin elicits embryo formation from unfertilized eggs in a sexual plant. PNAS.

[CR21] Cooke JEK, Eriksson ME, Junttila O (2012). The dynamic nature of bud dormancy in trees: Environmental control and molecular mechanisms. Plant Cell Environ.

[CR22] Dann M, Bellot S, Schepella S, Schaefer H, Tellier A (2017) Mutation rates in seeds and seed-banking influence substitution rates across the angiosperm phylogeny. bioRxiv preprint 156398.10.1101/156398

[CR23] Datta SC, Evenari M, Gutterman Y (1970). The heteroblasty of *Aegilops ovata*. Israel J Bot.

[CR24] de Casas RR, Donohue K, Venable DL, Cheptou P-O (2015). Gene-flow through space and time: dispersal, dormancy and adaptation to changing environments. Evol Ecol.

[CR25] Dyer AR (2017). The seed ecology of *Aegilops triuncialis*: linking trait variation to growing conditions. Seed Sci Res.

[CR26] Footitt S, Clay HA, Dent K, Finch-Savage WE (2014). Environment sensing in spring-dispersed seeds of a winter annual Arabidopsis influences the regulation of dormancy to align germination potential with seasonal changes. New Phytol.

[CR27] Footitt S, Müller K, Kermode AR, Finch-Savage WE (2015). Seed dormancy cycling in Arabidopsis: chromatin remodelling and regulation of *DOG1* in response to seasonal environmental signals. Plant J.

[CR28] Footitt S, Walley PG, Lynn JR, Hambidge AJ, Penfield S, Finch-Savage WE (2019). Trait analysis reveals *DOG1* determines initial depth of seed dormancy, but not changes during dormancy cycling that result in seedling emergence timing. New Phytol.

[CR29] Gianella M, Balestrazzi A, Pagano A, Müller JV, Kyratzis AC, Kikodze D, Canella M, Mondoni A, Rossi G, Guzzon F (2020). Heteromorphic seeds of wheat wild relatives show germination niche differentiation. Plant Biol.

[CR30] Gioria M, Pyšek P, Baskin CC, Carta A (2020). Phylogenetic relatedness mediates persistence and density of soil seed banks. J Ecol.

[CR31] Gremer JR, Venable DL (2014). Bet hedging in desert winter annual plants: Optimal germination strategies in a variable environment. Ecol Lett.

[CR32] Guzzon F, Orsenigo S, Gianella M, Müller JV, Vagge I, Rossi G, Mondoni A (2018). Seed heteromorphy influences seed longevity in *Aegilops*. Seed Sci Res.

[CR33] Haaland TR, Wright J, Ratikainen II (2020). Generalists versus specialists in fluctuating environments: a bet-hedging perspective. Oikos.

[CR34] Herman JJ, Spencer HG, Donohue K, Sultan SE (2013). How stable “should” epigenetic modifications be? Insights from adaptive plasticity and bet hedging. Evol.

[CR35] Herrera CM, Medrano M, Bazaga P (2014). Variation in DNA methylation transmissibility, genetic heterogeneity and fecundity-related traits in natural populations of the perennial herb *Helleborus foetidus*. Mol Ecol.

[CR36] Hopper KR, Rosenheim JA, Prout T, Oppenheim SJ (2003). Within-generation bet hedging: a seductive explanation?. Oikos.

[CR37] Horst JL, Venable LD (2017). Frequency-dependent seed predation by rodents on Sonoran Desert winter annual plants. Ecol.

[CR38] Hradilová I, Duchoslav M, Brus J, Pechanec V, Hýbl M, Kopecký P, Smržová L, Štefelová N, Vaclávek T, Bariotakis M, Machalová J, Hron K, Pirintsos S, Smýkal P (2019). Variation in wild pea (Pisum sativum subsp elatius) seed dormancy and its relationship to the environment and seed coat traits. PeerJ.

[CR39] Huang Z, Liu S, Bradford KJ, Huxman TE, Venable DL (2016). The contribution of germination functional traits to population dynamics of a desert plant community. Ecol.

[CR40] Hulme PE (1998). Post-dispersal seed predation and seed bank persistence. Seed Sci Res.

[CR41] Huxman TE, Barron-Gafford G, Gerst KL, Angert AL, Tyler AP, Venable LD (2008). Photosynthetic resource-use efficiency and demographic variability in desert winter annual plants. Ecol.

[CR42] Imbert E (2002). Ecological consequences and ontogeny of seed heteromorphism. Perspect Plant Ecol Evol Syst.

[CR43] Iriondo JM, Milla R, Volis S, Rubio de Casas R (2018). Reproductive traits and evolutionary divergence between Mediterranean crops and their wild relatives. Plant Biol.

[CR44] Jiang L, Wang L, Baskin CC, Tian CY, Huang ZY (2019). Maternal effects on seed heteromorphism: a dual dynamic bet hedging strategy. Seed Sci Res.

[CR45] Johnson JS, Cantrell RS, Cosner C, Hartig F, Hastings A, Rogers HS, Schupp EW, Shea K, Teller BJ, Yu X, Zurell D, Pufal G (2019) Rapid changes in seed dispersal traits may modify plant responses to global change. AoBP 11: plz020 10.1093/aobpla/plz020. 10.1093/aobpla/plz020 PMC6548345 31198528

[CR46] Johnston IG, Bassel GW (2018). Identification of a bet-hedging network motif generating noise in hormone concentrations and germination propensity in Arabidopsis. J R Soc Interface.

[CR47] Khanday I, Skinner D, Yang B, Mercier R, Sundaresan V (2019). A male-expressed rice embryogenic trigger redirected for asexual propagation through seeds. Nature.

[CR48] Kumar S (2017) Epigenetic control of apomixis: a new perspective of an old enigma. Adv Plants Agric Res 7:227-233. 10.15406/apar.2017.07.00243

[CR49] Lamont BB, Pausas JG, He T, Witkowski ETF, Hanley ME (2020). Fire as a selective agent for both serotiny and nonserotiny over space and time. Crit Rev Plant Sci.

[CR50] Lenser T, Tarkowská D, Novák O, Wilhelmsson PKI, Bennett T, Rensing SA, Strnad M, Theißen G (2018). When the BRANCHED network bears fruit: how carpic dominance causes fruit dimorphism in *Aethionema*. Plant J.

[CR51] Lepiniec L, Debeaujon I, Routaboul JM, Baudry A, Pourcel L, Nesi N, Caboche M (2006). Genetics and biochemistry of seed flavonoids. Annu Rev Plant Biol.

[CR52] Leverett LD, Jolls CL (2013). Cryptic seed heteromorphism in *Packera tomentosa* (Asteraceae): Differences in mass and germination. Plant Spec Biol.

[CR53] Li W, Liu X, Hanada A, Khan MA (2011) Effect of cold stratification, scarification and hormones on germination of dimorphic seeds of *Atriplex centralasiatica* under saline conditions. Seed Sci Technol 1: 82–92 10.15258/sst.2011.39.1.08

[CR54] Liu S, Bradford KJ, Huang Z, Venable DL (2020). Hydrothermal sensitivities of seed populations underlie fluctuations of dormancy states in an annual plant community. Ecology.

[CR55] Long RL, Gorecki MJ, Renton M, Scott JK, Colville L, Goggin DE, Commander LE, Westcott DA, Cherry H, Finch-Savage WE (2015). The ecophysiology of seed persistence: A mechanistic view of the journey to germination or demise. Biol Rev.

[CR56] Lu JJ, Tan DY, Baskin JM, Baskin CC (2013). Trade-offs between seed dispersal and dormancy in an amphi-basicarpic cold desert annual. Ann Bot.

[CR57] Martínez-Berdeja A, Stitzer M, Taylor M, Okada M, Ezcurra E, Runcie DE, Schmitt J (2020). Functional variants of *DOG1* control seed chilling responses and variation in seasonal life-history strategies in *Arabidopsis thaliana*. PNAS.

[CR58] Minow MAA, Colasanti J (2020). Does variable epigenetic inheritance fuel plant evolution?. Genome.

[CR59] Mitchell J, Johnston IG, Bassel GW (2017). Variability in seeds: Biological, ecological, and agricultural implications. J Exp Bot.

[CR60] Moncalvillo B, Méndez M, Iriondo JM (2019). Ecotypic differentiation reveals seed colour-related alkaloid content in a crop wild relative. Plant Biol.

[CR61] Mueller NG (2017). Documenting domestication in a lost crop (*Polygonum erectum* L.): evolutionary bet-hedgers under cultivation. Veg Hist Archaeobot.

[CR62] Nave M, Avni R, Ben-Zvi B, Hale I, Distelfeld A (2016). QTLs for uniform grain dimensions and germination selected during wheat domestication are co-located on chromosome 4B. Theor Appl Genet.

[CR63] Nichols BS, Leubner-Metzger G, Jansen VAA (2020). Between a rock and a hard place: adaptive sensing and site-specific dispersal. Ecol Lett.

[CR64] Niklas KJ, Cobb ED (2017). The evolutionary ecology (evo-eco) of plant asexual reproduction. Evol Ecol.

[CR65] Nilsson P, Tuomi J, Åström M (1996). Bud dormancy as a bet-hedging strategy. Am Nat.

[CR66] Nisar F, Gul B, Khan MA, Hameed A (2019). Heteromorphic seeds of coastal halophytes *Arthrocnemum macrostachyum* and *A. indicum* display differential patterns of hydrogen peroxide accumulation, lipid peroxidation and antioxidant activities under increasing salinity. Plant Physiol Bioch.

[CR67] O’dea RE, Noble DWA, Johnson SL, Hesselson D, Nakagawa S (2015). The role of non-genetic inheritance in evolutionary rescue: epigenetic buffering, heritable bet hedging and epigenetic traps. Environmental Epigenetics.

[CR68] Olofsson H, Ripa J, Jonzén N (2009). Bet-hedging as an evolutionary game: the trade-off between egg size and number. Proc R Soc Lond [Biol].

[CR69] Peters EM, Martorell C, Ezcurra E (2011). The effects of serotiny and rainfall-cued dispersal on fitness: Bet-hedging in the threatened cactus *Mammillaria pectinifera*. Popul Ecol.

[CR70] Philippi T (1993). Bet-hedging germination of desert annuals: variation among populations and maternal effects in *Lepidium lasiocarpum*. Am Nat.

[CR71] Philippi T, Seger J (1989). Hedging one’s evolutionary bets, revisited. Trends Ecol Evol.

[CR72] Renzi JP, Duchoslav M, Brus J, Hradilová I, Pechanec V, Václavek T, Machalová J, Hron K, Verdier J, Smýkal P (2020). Physical dormancy release in *Medicago truncatula* seeds is related to environmental variations. Plants.

[CR73] Rodrigues JCM, Koltunow AMG (2005). Epigenetic aspects of sexual and asexual seed development. Acta Biol Cracov Bot.

[CR74] Santini BA, Martorell C (2013). Does retained-seed priming drive the evolution of serotiny in drylands? An assessment using the cactus *Mammillaria hernandezii*. Am J Bot.

[CR75] Scholl JP, Calle L, Miller N, Venable DL (2020). Offspring polymorphism and bet hedging: a large-scale, phylogenetic analysis. Ecol Lett.

[CR76] Seger J, Brockmann HJ, Harvey PH, Partridge L (1987). What is Bet-Hedging. Oxford Surveys in Evolutionary Biology: 182–211.

[CR77] Simons AM (2009). Fluctuating natural selection accounts for the evolution of diversification bet hedging. Proc R Soc Lond [Biol].

[CR78] Simons AM, Johnston MO (2006). Environmental and genetic sources of diversification in the timing of seed germination: implication for the evolution of bet hedging. Evolution.

[CR79] Slatkin M (1974). Hedging one’s evolutionary bets. Nature.

[CR80] Soliman MI (2003). Genetic diversity of achene heteromorphism in Egyptian *Calendula micrantha* Tineo et Guss. Asian J Plant Sci.

[CR81] Springthorpe V, Penfield S (2015). Flowering time and seed dormancy control use external coincidence to generate life history strategy. ELife.

[CR82] Stadler MR, Eisen MB (2017). Atlas…t, patterns from every cell. Science.

[CR83] Starrfelt J, Kokko H (2012). Bet-hedging-a triple trade-off between means, variances and correlations. Biol Rev.

[CR84] Still DW, Bradford KJ (1997). Endo-beta-mannanase activity from individual tomato endosperm caps and radicle tips in relation to germination rates. Plant Physiol.

[CR85] Suter L, Widmer A (2013). Phenotypic effects of salt and heat stress over three generations in *Arabidopsis thaliana*. PLoS ONE.

[CR86] Tellier A (2018). Persistent seed banking as eco-evolutionary determinant of plant nucleotide diversity: novel population genetics insights. New Phytol.

[CR87] ten Brink H, Gremer JR, Kokko H (2020). Optimal germination timing in unpredictable environments: the importance of dormancy for both among- and within-season variation. Ecol Lett.

[CR88] Thurlby KAG, Wilson PG, Sherwin WB, Connelly C, Rossetto M (2012). Reproductive bet-hedging in a rare yet widespread rainforest tree, *Syzygium paniculatum* (Myrtaceae). Austral Ecol.

[CR89] Topham AT, Taylor RE, Yan D, Nambara E, Johnston IG, Bassel GW (2017). Temperature variability is integrated by a spatially embedded decision-making center to break dormancy in Arabidopsis seeds. Proc Natl Acad Sci USA.

[CR90] Vaerbak S, Andersen SB (2004). Genetic control of seed set linked and unlinked to flower heteromorphism in inbred lines of *Primula vulgaris* Hudson. Euphytica.

[CR91] van Dooren TJM, Silveira AB, Gilbault E, Jiménez-Gómez JM, Martin A, Bach L, Tisné S, Quadrana L, Loudet O, Colot V (2020). Mild drought in the vegetative stage induces phenotypic, gene expression, and DNA methylation plasticity in Arabidopsis but no transgenerational effects. J Exp Bot.

[CR92] Venable DL (1985). The evolutionary ecology of seed heteromorphism. Am Nat.

[CR93] Venable DL (2007). Bet hedging in a guild of desert annuals. Ecology.

[CR94] Verin M, Tellier A (2018). Host-parasite coevolution can promote the evolution of seed banking as a bet-hedging strategy. Evolution.

[CR95] Volis S (2014). Dormancy-related seed positional effect in two populations of an annual grass from locations of contrasting aridity. PLoS ONE.

[CR96] Volis S, Bohrer G (2013). Joint evolution of seed traits along an aridity gradient: seed size and dormancy are not two substitutable evolutionary traits in temporally heterogeneous environment. New Phytol.

[CR97] Wang L, Dong M, Huang Z (2010). Review of research on seed heteromorphism and its ecological significance. Chinese J Plant Ecol.

[CR98] Wilhelmsson PKI, Chandler JO, Fernandez-Pozo N, Graeber K, Ullrich KK, Arshad W, Khan S, Hofberger JA, Buchta K, Edger PP, Pires JC, Schranz ME, Leubner-Metzger G, Rensing SA (2019). Usability of reference-free transcriptome assemblies for detection of differential expression: a case study on *Aethionema arabicum* dimorphic seeds. BMC Genom.

[CR99] Xu Y, Liu R, Sui N, Shi W, Wang L, Tian C, Song J (2016). Changes in endogenous hormones and seed-coat phenolics during seed storage of two *Suaeda salsa* populations. Aust J Bot.

[CR100] Xu Y, Zhao Y, Duan H, Sui N, Yuan F, Song J (2017). Transcriptomic profiling of genes in matured dimorphic seeds of euhalophyte *Suaeda salsa*. BMC Genomics.

[CR101] Zhao Y, Ma Y, Li Q, Yang Y, Guo J, Song J (2018). Utilisation of stored lipids during germination in dimorphic seeds of euhalophyte *Suaeda salsa*. Funct Plant Biol.

[CR102] Zinsmeister J, Leprince O, Buitink J (2020). Molecular and environmental factors regulating seed longevity. Biochem J.

